# Cyathostomin resistance to moxidectin and combinations of anthelmintics in Australian horses

**DOI:** 10.1186/s13071-021-05103-8

**Published:** 2021-12-04

**Authors:** Ghazanfar Abbas, Abdul Ghafar, John Hurley, Jenni Bauquier, Anne Beasley, Edwina J. A. Wilkes, Caroline Jacobson, Charles El-Hage, Lucy Cudmore, Peter Carrigan, Brett Tennent-Brown, Charles G. Gauci, Martin K. Nielsen, Kristopher J. Hughes, Ian Beveridge, Abdul Jabbar

**Affiliations:** 1 Melbourne Veterinary School, Faculty of Veterinary and Agricultural Sciences, The University of Melbourne, Werribee, VIC Australia; 2 Swettenham Stud, Nagambie, VIC Australia; 3 School of Agriculture and Food Sciences, University of Queensland, Gatton, QLD Australia; 4 School of Animal and Veterinary Sciences, Charles Sturt University, Wagga Wagga, NSW Australia; 5 Centre for Animal Production and Health, Murdoch University, Murdoch, WA Australia; 6 Scone Equine Hospital, Scone, NSW Australia; 7 Department of Veterinary Science, M.H. Gluck Equine Research Center, University of Kentucky, Lexington, KY USA

**Keywords:** Australian thoroughbred horses, Cyathostomins, Egg reappearance period, Moxidectin, Resistance, FECRT

## Abstract

**Background:**

Cyathostomins are the most important and common parasitic nematodes of horses, with > 50 species known to occur worldwide. The frequent and indiscriminate use of anthelmintics has resulted in the development of anthelmintic resistance (AR) in horse nematodes. In this study we assessed the efficacy of commonly used anthelmintics against cyathostomins in Australian thoroughbred horses.

**Methods:**

Two drug efficacy trials per farm were conducted on two thoroughbred horse farms in the state of Victoria, Australia. In the first trial, the horses on Farm A were treated with single and combinations of anthelmintics, including oxfendazole (OFZ), abamectin (ABM), abamectin and morantel (ABM + MOR), moxidectin (MOX) and oxfendazole and pyrantel (OFZ + PYR), at the recommended doses, whereas the horses on Farm B only received MOX, at the recommended dose. The faecal egg count reduction test (FECRT) was used to determine the efficacy and egg reappearance period (ERP) of anthelmintics. Based on the results of the first trial, the efficacies of MOX and a combination of ABM + MOR were reassessed to confirm their activities against cyathostomins.

**Results:**

Of the five anthelmintic products tested on Farm A, resistance against OFZ, ABM and OFZ + PYR was found, with efficacies of − 41% (− 195% lower confidence limit [LCL]), 73% (60% LCL) and 82% (66% LCL) at 2 weeks post-treatment, respectively. The FECRT showed high efficacies of MOX and ABM + MOR (100%) at 2 week post-treatment and shortened ERPs for these anthelmintics (ABM + MOR: 4 weeks; MOX: 5 weeks). Resistance to MOX was found on Farm B, with a reduced efficacy of 90% (70% LCL) and 89% (82% LCL) at 2 weeks post-treatment in trials one and two, respectively.

**Conclusions:**

This study provides the first evidence of MOX- and multidrug-resistant (ABM and combinations of anthelmintics) cyathostomins in Australia and indicates the need for continuous surveillance of the efficacy of currently effective anthelmintics and large-scale investigations to assess the ERP for various anthelmintics.

**Graphical Abstract:**

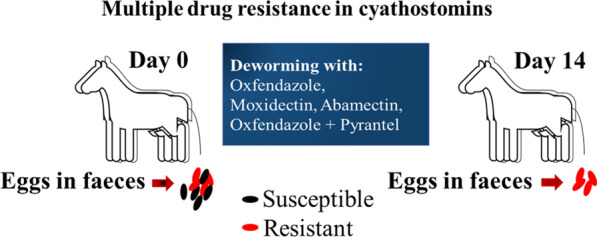

## Background

Small strongyles (Strongylida: Cyathostominae), also known as cyathostomins, are the most important and common parasitic nematodes of horses, with more than 50 species known to occur worldwide [1, 2]. Cyathostomins have a direct life-cycle, with horses becoming infected by ingesting third-stage (L3) infective larvae while grazing and the infective larvae then developing into adult male and female worms in the large intestine. The majority of cyathostomin infections are subclinical, while clinical manifestations of larval cyathostominosis (i.e. synchronous emergence of fourth-stage larvae from the intestinal wall) include weight loss, colic, pyrexia, diarrhea and subcutaneous oedema accompanied by marked hypoproteinemia, with a case fatality of up to 50% in horses of age ≤ 6 years [3, 4].

Control of cyathostomins in horses has traditionally relied on interval-based deworming using three classes of anthelmintics, benzimidazoles (BZs), tetrahydropyrimidines (THPs) and macrocyclic lactones (MLs) [5]. However, the frequent and indiscriminate use of anthelmintics has resulted in the development of anthelmintic resistance (AR) in nematodes infecting horses [5]. Anthelmintic resistance against BZs and THPs is widespread and well-established in cyathostomins, whereas sporadic accounts of resistance or reduced egg reappearance periods (ERP) against MLs (ivermectin [IVM] and moxidectin [MOX]) have been reported from various parts of the world [6–8]. For example, ML resistance in cyathostomins has been reported either in a single horse [9] or in a group of horses at the farm level, based on drug efficacy trials [8, 10–15]. In addition, multiple studies have reported reduced ERP for cyathostomins after administration of IVM or MOX [11, 13–22].

Due to the growing concern of resistance against individual anthelmintic drugs and/or classes, Barnes et al. [23] and Leathwick [24] used computer simulation modelling, with the results suggesting that combinations of ≥ 2 anthelmintics with similar nematocidal spectrums from different classes could delay the development of resistance. This alternative approach was based on the successful control of resistant worms of sheep using combinations of anthelmintics before resistance levels became too high [25, 26]. Although a combination of anthelmintics is now routinely used in some countries to control horse nematodes, limited information is available on the efficacy of such anthelmintic regimens against cyathostomins. Kaplan et al. [27] recently reported > 90%, > 95% and > 99% efficacies of the combination oxibendazole and pyrantel (OBZ + PYR) on 11, 9 and 6 of 11 horse farms, respectively. Notably, the individual efficacies of these drugs were < 90% [27]. Similarly, in another trial, an additive effect was observed for the efficacy of the OBZ + PYR combination against horse cyathostomins in the first of the four consecutive treatments, whereas the individual efficacies of both drugs were much lower [28]. In addition, a recent simulation-based study demonstrated that the use of a combination of anthelmintics could be helpful in delaying the development of AR in cyathostomins, despite one of the active ingredients exhibiting lower efficacy [29].

Although there are numerous reports of AR and reduced ERP in cyathostomins of horses from various parts of the world, little is known about the status of resistance against MLs, particularly MOX, and combinations of anthelmintics commonly used in Australian horses. Edward and Hoffmann [9] reported a suspected case of IVM resistance in cyathostomins based on post-treatment faecal egg counts (FEC). Subsequently, Beasley et al. [22] reported reduced ERP for MOX (12 weeks) and IVM (6 weeks) against cyathostomins on one and two properties, respectively. These findings instigated a regular surveillance of the efficacy and ERP for commonly used anthelmintics in Australian horses. Therefore, the aim of this study was to assess the efficacy and ERP of commonly used anthelmintics on two thoroughbred horse farms in Australia.

## Methods

### Selection of horse farms

The following selection criteria were used to enroll the farms in the study: (i) horses had not been dewormed in the last 8–10 weeks; (ii) a confirmation that the FEC of an individual horse was ≥ 45 eggs per gram (EPG) of faeces; and (iii) there was a known history of anthelmintic usage on the farm in the last 5 years (2015–2020). Horses selected for the study were assigned to treatment or control groups using simple randomisation. The control groups were selected to observe any natural variation in FECs of untreated horses during the study period.

This study was conducted during 2020–2021 on two thoroughbred horse farms (designated as A and B) in the state of Victoria, Australia where resident veterinarians were interested in participating. Farm A is located 200 km north of Melbourne and has approximately 600 horses. Horses of all ages (adults, yearlings and weanlings) are dewormed based on average group FEC (i.e. 10 faecal samples are randomly collected from each paddock and a FEC is performed on each sample). If the average EPG of faeces exceeds 500 EPG, all horses in the paddock are dewormed and the dose of anthelmintic is calculated according to herd-estimated average body weight in each age category. IVM and abamectin (ABM) and various anthelmintic combinations (oxfendazole and pyrantel [OFZ + PYR] and ABM and morantel [ABM + MOR]) have been used in the last 5 years (2015–2020), while a combination of ABM and MOR has been used for all age groups of horses in the last year (2019). Continuous grazing (i.e. set stocking) is used at the farm and horses are not moved out of the paddocks.

Farm B is located 110 km east of Melbourne and has approximately 60 thoroughbred horses. Adult horses on this farm are dewormed based on FEC surveillance: horses with a FEC > 500 EPG are dewormed. On this farm, young horses are dewormed routinely every 8–10 weeks, with the dose of anthelmintic for an individual horse calculated based on actual body weight. In the last year (2019), MOX was used to deworm all age groups of horses. On Farm B, alternate grazing is used, and horses are periodically moved from one paddock to the other and replaced with cattle and alpacas.

### Anthelmintic treatment and sample collection

Prior to the start of the study, FECs were performed at both farms to ascertain the worm egg count threshold required for the study. Anthelmintic dosage was calculated based on the individual horse body weight (Farm B) or weight of the heaviest animal within a treatment group (Farm A) and was administered as per the manufacturers’ recommendations. The administration of anthelmintics and the collection of faecal samples (directly from the rectum of the horses where possible) were performed by the resident farm veterinarians in the presence of the authors.

In the first trial conducted on Farm A, 30 weanlings (aged 7–8 months; male = 12; female =  18) were selected and randomly divided into six groups (5 animals in each group) after fulfilling the inclusion criteria as outlined above. Five groups were treated with a single or a combination of anthelmintics, including OFZ, ABM, MOX, OFZ + PYR and ABM + MOR at recommended doses while the sixth group was an untreated control group (Table 1). For MOX, a formulation with a combination of praziquantel (PZQ) and MOX was used. Given PZQ has no nematocidal activity, we designated this combination as MOX throughout the paper. Faecal samples were collected on day 0 (immediately prior to treatment) and then weekly (apart from week 4) until 6 weeks post-treatment (where no resistance was detected in the second or third weeks). On Farm B, 14 yearlings (aged 12–13 months; male = 3; female =  11) were selected and randomly divided into two groups (7 animals in each group). One group was treated with MOX and the second group was an untreated control group.

**Table 1:** Details of anthelmintic drugs used in this study

Group	Trial(s)	Drugs	Active ingredient	Dose (per kg body weight)
Farm A	1	AMMO Rotational Wormer® (Ceva Animal Health Pty Ltd, Glenorie, NSW, Australia)	Oxfendazole (OFZ)	10 mg
	1	MecWorma & Bot® (International Animal Health Products, Huntingwood, NSW, Australia)	Abamectin (ABM)	0.2 mg
	1, 2	Strategy-T® (Virbac Australia Pty Ltd, Peakhurst, NSW, Australia)	Oxfendazole and pyrantel (OFZ + PYR)	10 mg oxfendazole + 6.6 mg pyrantel base
	1, 2	Equest® Plus Tape (Zoetis Australia Pty Ltd, Rhodes, NSW, Australia)	Moxidectin and praziquantel (MOX + PZQ)	0.4 mg
	1	MecWorma & Tape® (International Animal Health Products)	Abamectin and morantel tartrate (ABM + MOR)	54 mg
	2	AMMO Allwormer Wormer® (Ceva Animal Health Pty Ltd)	Abamectin and morantel tartrate (ABM + MOR)	54 mg
	1, 2	Untreated control	–	
Farm B	1, 2	Equest® Plus Tape (Zoetis Australia, Pty Ltd)	Moxidectin and praziquantel (MOX + PZQ)	0.4 mg
	1, 2	Untreated control	–	

Based on the findings of trial 1 on Farm A, the efficacies of MOX and ABM + MOR (Table 1) were re-tested in 15 weanlings (aged 5–6 months; male = 7; female =  8) 8 months after the first trial. For this purpose, the weanlings were divided into three groups (5 animals in each group), and two groups were treated with anthelmintics (MOX or ABM + MOR) while the third group was as an untreated control group. Similarly, a second trial was conducted on Farm B in which only the efficacy of MOX was re-tested in a group of 10 weanlings (aged 7–8 months; male = 3; female  =  7) 9 months after the first trial; the horses were divided into two groups (5 animals in each group), and one group was treated with MOX while the second group was an untreated control group.

### Faecal egg counts

Faecal egg counts were carried out within 48 to 96 h of collection of faeces using the Modified McMaster technique [30]. Briefly, 4 g of faeces was mixed with 4 ml of water to make a homogeneous slurry which was then mixed with 52 ml of sucrose solution (specific gravity = 1.27; www.csrsugar.com.au) and homogenised using a spatula. Following homogenisation, a sample (volume = 1 ml) was pipetted into two chambers of a Whitlock egg counting slide (www.whitlock.com.au). After 10 min, eggs were counted using a compound light microscope. A multiplication factor of 15 for this method was applied to calculate the number of eggs per gram.

### FEC reduction test and ERP

The percentage FEC reduction (%FECR) was calculated each week to assess the efficacy of the anthelmintics tested, and resistance to a particular anthelmintic was declared as per the guidelines of the American Association of Equine Practitioners (AAEP) [31]. Briefly, group-based %FECR was calculated (utilising the equation below) using the arithmetic group mean FECs at pre-treatment and 2 weeks post-treatment to declare the efficacy of an anthelmintic in the group. The %FECR for each treatment group along with 95% uncertainty interval was also analysed using the Bayesian hierarchical model in an online web interface [32]:

$$\mathrm{FECR }\%=\frac{\mathrm{EPG }\left(\mathrm{pre}-\mathrm{treatment}\right)-\mathrm{EPG }(\mathrm{post}-\mathrm{treatment})}{\mathrm{EPG }(\mathrm{pre}-\mathrm{treatment})} \times 100$$

Given that equine-specific criteria are yet to be established to define the presence of resistance to individual MLs and drug combinations, an efficacy of %FECR of > 95% for MLs/drug combinations and > 90% for BZs/THPs was used. Additionally, 95% lower confidence limits (LCLs) of 90% and 80% were selected for classifying resistance to MLs/drug combinations and BZs/THPs, respectively, as per the guidelines of the World Association for the Advancement of Veterinary Parasitology (WAAVP) [33]. The 95% confidence intervals were calculated based on the delta method as described by Levecke et al. [34]. Resistance to a particular anthelmintic was confirmed if it failed to meet both thresholds, while a result where only one of the two criteria was met was considered to be suspected resistance. Likewise, there is no consensus on the calculation/interpretation of ERP in horses and no guidelines exist for setting a cut-off for the ERP of combination products. For this study, we adopted the standard where ERP is defined as the time elapsed from day 0 to when %FECR returns to < 90% [16].

### Use of PCR for nematode identification

In order to rule out the presence of large strongyles (i.e. *Strongylus* spp.), a PCR targeting the second internal transcribed spacer (ITS-2) of the nuclear ribosomal DNA was performed as described previously [35]. DNA was extracted from pre- and post-treatment pooled faecal samples from each treated and untreated group using the DNeasy PowerSoil Pro Kit (Qiagen, Hilden, Germany) according to the manufacturer’s protocol. PCRs were performed in a final volume of 25 µl using the *Strongylus* genus-specific primers NC4_F (5′-TGAAATTKGAACGAAT-3′) and NC2_R (5′- TTAGTTCTTTTCCTCCGCT-3′) in a T100 thermal cycler (BioRad Laboratories, Hercules, CA, USA) using the same conditions as described by Campbell et al. [35]. Known positive (genomic DNA of *Strongylus edentatus*) and negative (Milli-Q H_2_O) controls were included in each PCR run. Aliquots (5 μl) of individual amplicons were analysed on 1.5% (w/v) agarose gels in Tris–Borate–EDTA buffer, stained with GelRed (Biotium, Fremont, CA, USA) and visualised using a GelDoc system (BioRad Laboratories).

## Results

### Anthelmintic efficacy

**Fig. 1:**
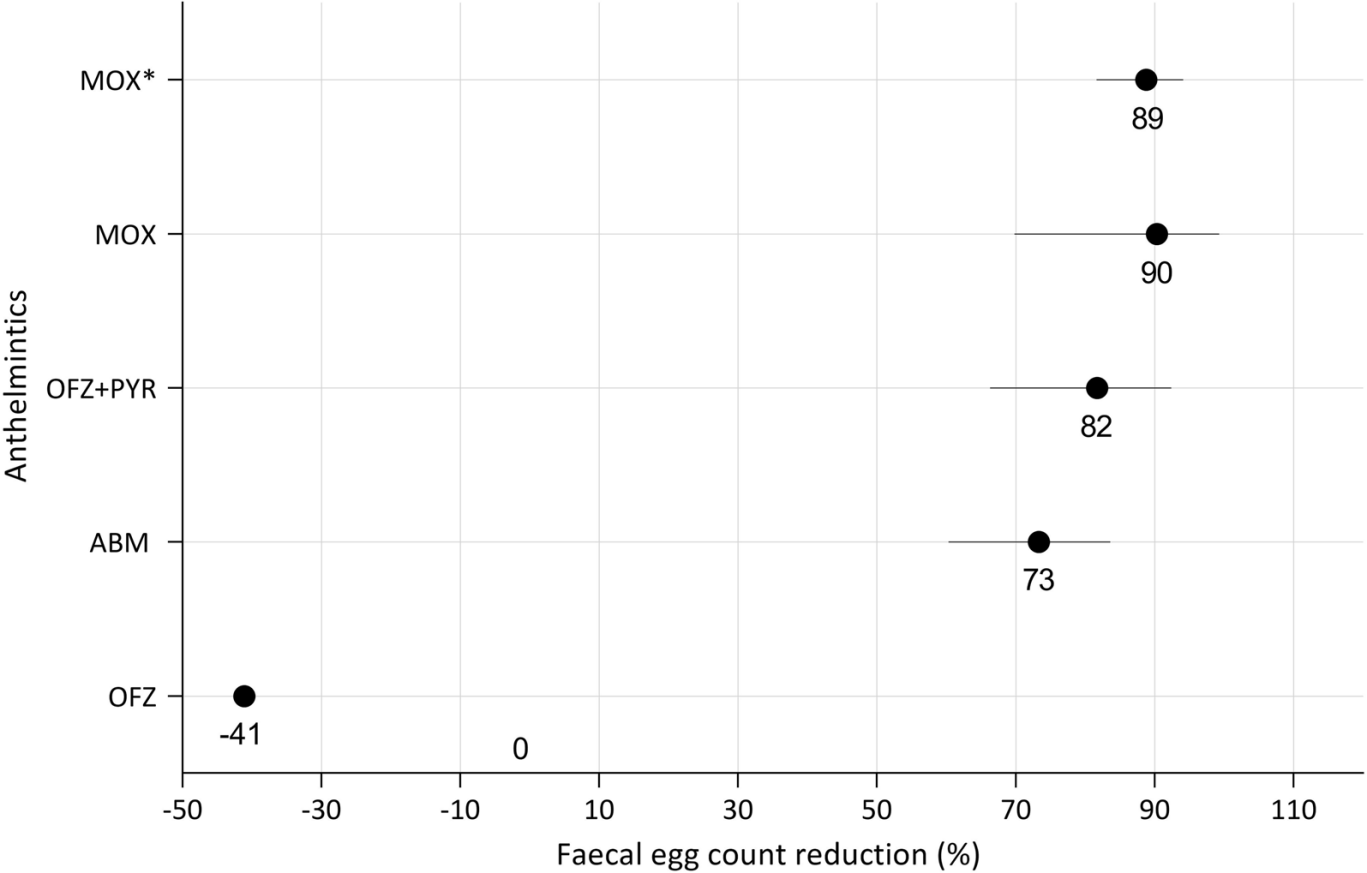
Efficacy of anthelmintics against cyathostomins on Farms A and B, based on 2-weekly post-treatment faecal egg counts. ABM, OFZ and OFZ + PYR were tested on Farm A while MOX was tested on Farm B. Each circle shows the percentage of the faecal egg count reduction (%FECR) for each anthelmintic at 2 weeks post-treatment while each horizontal black line shows the upper and lower 95% confidence intervals. Lower confidence limits of < 0 were considered to be 0. The asterisk indicates the anthelmintic used in the second trial. *ABM* Abamectin, *MOX* moxidectin, *OFZ* oxfendazole, *OFZ + PYR* oxfendazole and pyrantel combination

**Fig. 2:**
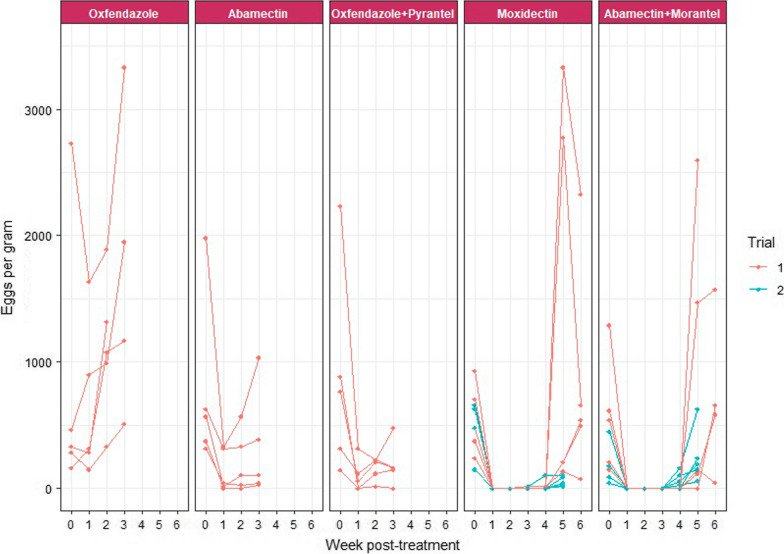
Weekly faecal egg counts (eggs per gram of faeces) of cyathostomins in individual horses at day 0 up to 6 weeks post-deworming for each anthelmintic used in both trials 1 and 2 at Farm A. Each circle represents the value of an individual faecal egg count per week

The weanlings on Farm A had pre-treatment FECs of cyathostomins ranging from 150 to 2730 EPG and from 45 to 630 EPG for the first and second trials, respectively. In the first trial, resistance to OFZ (− 41% FECR; − 195% LCL), ABM (73% FECR; 60% LCL) and OFZ + PYR (82% FECR; 66% LCL) was observed 2 weeks post-treatment (Fig. 1). The %FECR based on Bayesian hierarchical model analysis using group mean FECs pre- and post-treatment values resulted in the same efficacy as calculated by the method described in the AAEP parasite control guidelines. However, the former method also calculated the CI where efficacy was 100% (Table 2). The FEC of the majority of animals in each treatment group did not return to 0 EPG even by 1 week post-treatment. A consistent increase in individual horse EPG was noted in the following weeks for all anthelmintics tested, with the exception of MOX and ABM + MOR where cyathostomin eggs reappeared in faeces at ≥ 3 weeks post-treatment (Fig. 2).

**Table 2:** Cyathostomin faecal egg counts and percentage faecal egg count reduction at 2 weeks post-treatment with 95% confidence intervals

Treatment group	Farm	Trial	N*	FECs pre-treatment (EPG)		FECs post-treatment (EPG) week 2		N**	%FECR (95% CI) by AAEP method	%FECR (95% CI) Bayesian hierarchical model
				Mean	Range	Mean	Range			
Oxfendazole	A	1	5	795	165–2730	1122	330–1890	5	− 41 (− 195 to − 57)	0 (0–5)
Abamectin	A	1	5	774	315–1980	207	0–570	4	73 (60–84)	73 (65–79)
Moxidectin	A	1	5	546	240–930	0	–	0	100	100 (98–100)
Abamectin + morantel	A	1	5	561	150–1290	0	–	0	100	100 (98–100)
Oxfendazole + pyrantel	A	1	5	870	150–2235	159	15–225	5	82 (66–92)	82 (76–86)
Moxidectin	B	1	7	1530	75–3480	148	0–675	5	90 (70–99)	90 (88–93)
Moxidectin	A	2	5	480	150–630	0	–	0	100	100 (98–100)
Abamectin + morantel	A	2	5	162	45–450	0	–	0	100	100 (94–100)
Moxidectin	B	2	5	1041	150–1455	117	75–165	5	89 (82–94)	89 (85–92)

*AAEP *American Association of Equine Practitioners,* CI* confidence interval, *EGP* eggs per gram, *FEC* faecal egg count, *FECR* faecal egg count reduction, N* number of horses in group, N** number of horses shedding eggs within a group 2 weeks post-treatment

On Farm B, the initial FEC of cyathostomins ranged from 75 to 3480 EPG and from 150 to 1455 EPG for yearlings and weanlings, respectively. Resistance to MOX was found in both trials, with FECR of 90% (70% LCL) and 89% (82% LCL) 2 weeks post-treatment in the first and second trials, respectively (Fig. 1).

The FECs of cyathostomins of horses included in the control group were found to be consistently positive throughout the study period in both trials, with no sign of a clinical disease.

### ERP for MOX and ABM + MOR

**Fig. 3:**
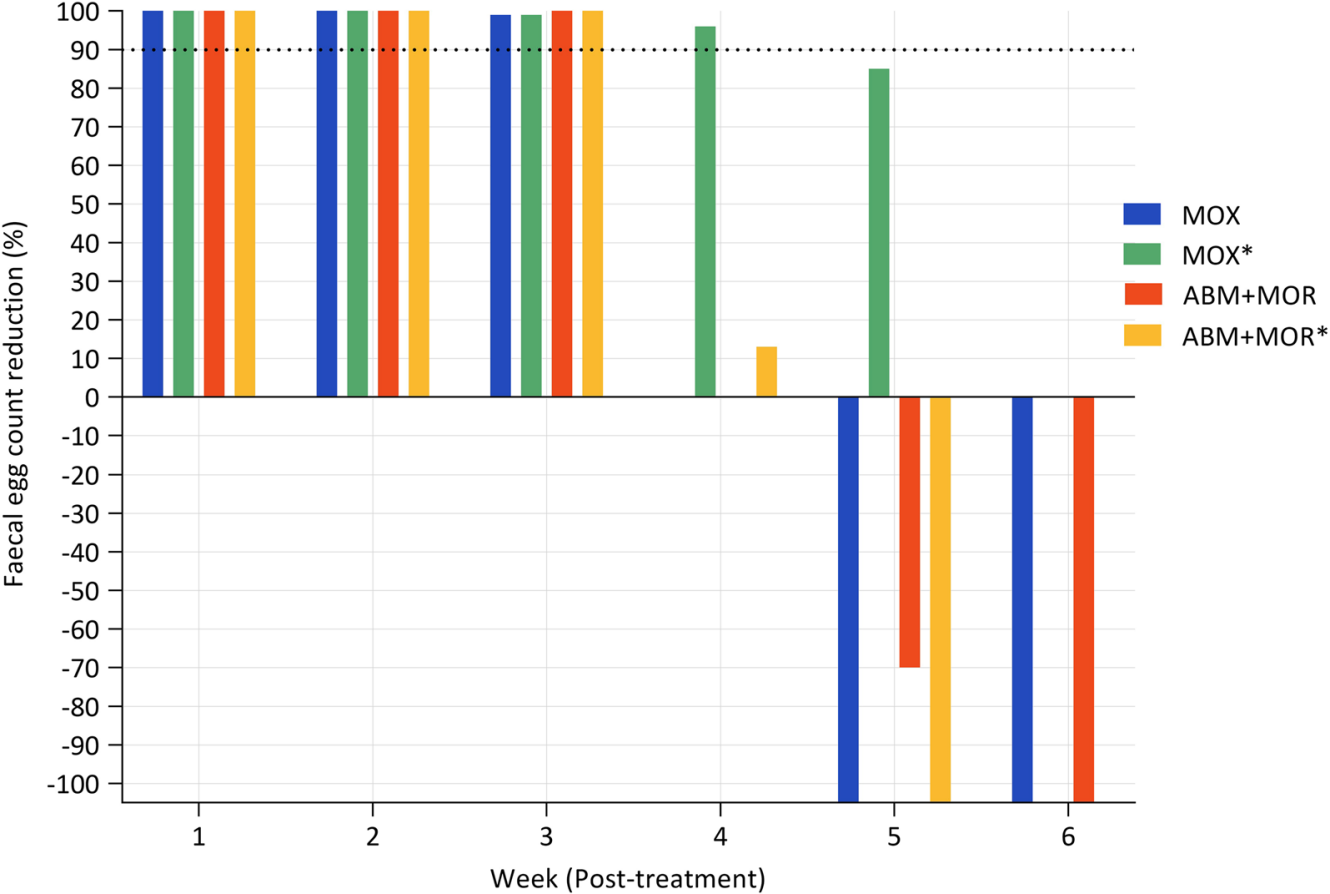
The efficacy (%FECR) of MOX and the ABM + MOR combination at Farm A up to 5 and 6 weeks post-treatment in trials 2 and 1, respectively. The 90% FECR threshold for defining the egg reappearance period is indicated with a black dotted line. The asterisk indicates the anthelmintic used in the second trial. *ABM + MOR* Abamectin and morantel combination

Out of five anthelmintic products tested in trial one on Farm A, only MOX and ABM + MOR resulted in 100% FECR 2 weeks post-treatment; therefore, we determined the ERP for these two anthelmintic products only. Three weeks post-treatment, one of the horses in the MOX-treated group tested positive for cyathostomin eggs; however, the group %FECR remained high at 99% and 100% for MOX and ABM + MOR, respectively. Subsequently, the sampling frequency was reduced to once every 2 weeks during trial 1 due to the labour required for the collection of faecal samples. At 5 weeks post-treatment, a sharp decrease in the efficacies of both MOX and ABM + MOR was observed which continued into the sixth week (Fig. 3). Hence, the ERP for both MOX and ABM + MOR was considered to be 5 weeks in the first trial. In the second trial, post-treatment weekly %FECR data revealed that the ERP for ABM + MOR and MOX was 4 and 5 weeks, respectively (Fig. 3).

### Identification of parasites

PCR amplicons revealed no band on 1.5% agarose gels in any of the pre- and post-treatment pooled faecal samples for all groups of horses while the positive control verified an expected amplicon size of approximately 370 bp, confirming that infections did not involve large strongyles.

## Discussion

This is the first report of resistance in cyathostomins to ABM. This study not only provides evidence of resistance in cyathostomins to single anthelmintics (ABM, OFZ and MOX) but is the first account of multidrug resistance in cyathostomins to treatment with a combination of anthelmintics (OFZ + PYR) on a single farm. The observed efficacies of OFZ, ABM, MOX and OFZ + PYR at 2 weeks post-treatment (− 41%, 73%, 90 and 89% and 82%, respectively), were substantially lower than those used for declaring AR as outlined in the AAEP guidelines [31]. However, the efficacies of MOX and ABM + MOR were 100% at 2 weeks post-treatment in one trial, but those of both drugs decreased below the ERP cut-off limits within 4–5 weeks post-treatment.

This study presents the first report of AR and markedly reduced ERP for MLs (5 weeks for MOX) and a drug combination (4 weeks for ABM + MOR) in cyathostomins in Australian horses while the manufacturer claimed ERP of at least 14 weeks for MOX in Australia [36]. Previously, only one study has reported reduced ERP of 6 and 12 weeks for IVM and MOX, respectively, in cyathostomins [22]. Since the introduction of MOX formulations for use in horses, reduced efficacy in treating cyathostomins in donkeys was first reported in an abstract published in a conference proceedings [37], in which the mean %FECR in one of the treatment groups was 87% and 31% on 14 and 25 days post-treatment, respectively. However, this lower efficacy could be due to the off-label use of MOX (formulation for intramuscular use in cattle) which might have affected the pharmacokinetics of the drug [38]. A recent study in Brazilian military horses reported resistance in cyathostomins to MOX; however, in this study anthelmintics were administered every 30 to 90 days and the study was started 30 days following the last treatment, with the results possibly representing a selection bias for MOX-resistant cyathostomins [39]. In comparison, in our study, MOX registered for use in horses was used at the recommended dose rate, and selected horses had not been dewormed in the 8–10 weeks prior to the start of both trials. However, resistance to ABM and MOX was confirmed in weanlings and yearlings at the same farms. These resistant worms could have evolved as a result of intrinsic farm factors, such as selection pressure, frequent mutation events resulting in the recurrent appearance of pre-existing alleles in cyathostomins [40] or importation of such genotypes through the introduction of new horses onto the farms. Farm A is a large thoroughbred breeding farm where > 200 mares arrive for breeding each year from various local and interstate localities. Farm B routinely imports horses from other countries. Such horizontal transfer of resistant worms was recently demonstrated in a study assessing ML efficacy in cyathostomins on a US horse farm where resistance to IVM and MOX was detected in cyathostomins in yearlings recently imported from Ireland, suggesting the importation of resistant cyathostomins from Ireland to the USA [8].

Among various predisposing factors for the development of AR in cyathostomins in this study, the frequency of deworming is likely a reason for the resistance to MOX as this anthelmintic was routinely used on Farm B for at least 1 year, with an interval of 8–10 weeks between treatments. In previous studies, the frequent use of anthelmintics was found to be associated with the development of AR [41, 42]. Another plausible factor for the development of resistance in cyathostomins to MOX (as compared to other MLs) is the high efficacy of this drug against larvae [43–45], minimising refugia and leading to greater selection pressure for resistant worms. A recent simulation-based study found that climate, season and the number of treatments per year were key factors favouring the development of AR in cyathostomins [42]. Given Australia’s diverse climatic zones with profound seasonal variations and the recommended interval for the deworming of horses being common across all of these zones, larger scale studies are needed to assess the prevalence of AR in MLs, such as ABM and MOX, which are considered the last hope of ‘perceived’ effective anthelmintics against cyathostomins in horses.

The resistance to OFZ in cyathostomins found in this study is consistent with the results of previous studies which report widespread and well-established resistance patterns in cyathostomins against BZs [6, 46–49]. The resistance to OFZ might have contributed to the reduced efficacy of the OFZ + PYR combination (82% FECR) at 2 weeks post-treatment in the current study. Although resistance was found to both OFZ and OFZ + PYR, the combination of anthelmintics yielded increased efficacy compared to OFZ alone. This additive efficacy could be due to preserved efficacy of PYR which was not tested alone due to its unavailability in Australia. A similar phenomenon was observed previously by Scare et al. [28] who reported enhanced efficacy (but still below effective limits: 76.6% FECR) of the OBZ + PYR combination against cyathostomins whereas the individual anthelmintic efficacies were 66.7% and 63.3%, respectively. However, the maximum efficacy achieved was not sustained in successive trials, suggesting that combination therapy against a double resistant cyathostomin population is not sustainable.

For a timely diagnosis of AR, assessment of ERP is considered to be an early indicator [5]. In this study, we found reduced ERP for MOX and ABM + MOR on Farm A. Shortened ERP for cyathostomins following ML treatments is widely reported in the literature. For example, an ERP of 4 weeks for cyathostomins following treatment with MOX has been reported in the USA [50]. Likewise, a European study reported an ERP of 6–8 weeks after treatment with MOX [51]. The longer ERP for MOX, in comparison to other ML drugs, is likely due to greater efficacy against various developmental stages of cyathostomins. However, in a recent study, the efficacy of MOX against the immature stages of cyathostomins (late L3/L4) was reduced, resulting in a decreased ERP [50], with the development of cross-resistance among MLs possibly contributing to the reduction in efficacy. The phenomenon of cross-resistance has been shown in *Haemonchus contortus* (a stomach nematode of sheep and goats), when rodents were infected with IVM-resistant and -susceptible strains of the parasite and then treated with MOX [52]. The authors found that MOX achieved an efficacy of ≤ 47.2% against an IVM-resistant strain at a dose that invariably killed ≥ 98% of an IVM-susceptible strain, suggesting that worms resistant to one ML may likely be resistant to another ML [52]. More recently, resistance to MOX was found in cyathostomins following confirmation of IVM resistance in a group of imported horses [8]. The resistance to ABM alone found in the current study could have not only affected the efficacy of a related anthelmintic, i.e. MOX, but possibly also led to reduced efficacy of ABM + MOR on Farm A.

Although the efficacies of some anthelmintic products were re-assessed to ascertain the presence or absence of resistance, the findings of this study should be interpreted keeping in view possible limitations, such as (i) a small number of horses per treatment group and (ii) a low FEC threshold for the selection of animals. Furthermore, owing to the unavailability of single formulations of MOR and PYR for equines in Australia, we only tested available combinations of these drugs. Therefore, future studies should test the efficacy of single anthelmintics along with combinations, where available, using larger numbers of animals per treatment group and a higher FEC threshold.

## Conclusion

This study provides the first report of resistance to ABM, MOX and a combination of anthelmintics in cyathostomins. MOX is arguably the last effective anthelmintic to manage cyathostomins in horses; however resistance was detected on more than one occasion in this study. The detection of cyathostomin resistance and/or reduced ERP to MLs (when used as a single anthelmintic and in combination) are concerning, and warrant the use of alternative worm control strategies. Further field studies involving a greater number of horses per group are required to assess the prevalence of resistance to single and multiple anthelmintics in cyathostomin populations. Consensus on FEC-based methods and interpretation of the detection of reduced drug efficacy/resistance, i.e. FECRT and ERP, are required to facilitate the monitoring, reporting and comparison of data between studies.

## Data Availability

All data generated or analysed during this study are included in this published article.
